# Comparative Analyses of QTLs Influencing Obesity and Metabolic Phenotypes in Pigs and Humans

**DOI:** 10.1371/journal.pone.0137356

**Published:** 2015-09-08

**Authors:** Sameer D. Pant, Peter Karlskov-Mortensen, Mette J. Jacobsen, Susanna Cirera, Lisette J. A. Kogelman, Camilla S. Bruun, Thomas Mark, Claus B. Jørgensen, Niels Grarup, Emil V. R. Appel, Ehm A. A. Galjatovic, Torben Hansen, Oluf Pedersen, Maryse Guerin, Thierry Huby, Philipppe Lesnik, Theo H. E. Meuwissen, Haja N. Kadarmideen, Merete Fredholm

**Affiliations:** 1 Department of Veterinary Clinical and Animal Sciences, Faculty of Health and Medical Sciences, University of Copenhagen, Copenhagen, Denmark; 2 The Novo Nordisk Foundation Center for Basic Metabolic Research, Faculty of Health and Medical Sciences, University of Copenhagen, Copenhagen, Denmark; 3 INSERM UMR_S 1166, Integrative Biology of Atherosclerosis Team, F-75013, Paris, France; 4 Sorbonne Universités UPMC Univ Paris 06 UMR_S 1166, Integrative Biology of Atherosclerosis Team, F-75013, Paris, France; 5 Institute of Cardiometabolism and Nutrition (ICAN), Pitié-Salpêtrière Hospital, 75013, Paris, France; 6 Institute of Animal and Agricultural Sciences, Norwegian University of Life Sciences, Ås, Norway; University of Lleida, SPAIN

## Abstract

The pig is a well-known animal model used to investigate genetic and mechanistic aspects of human disease biology. They are particularly useful in the context of obesity and metabolic diseases because other widely used models (e.g. mice) do not completely recapitulate key pathophysiological features associated with these diseases in humans. Therefore, we established a F2 pig resource population (n = 564) designed to elucidate the genetics underlying obesity and metabolic phenotypes. Segregation of obesity traits was ensured by using breeds highly divergent with respect to obesity traits in the parental generation. Several obesity and metabolic phenotypes were recorded (n = 35) from birth to slaughter (242 ± 48 days), including body composition determined at about two months of age (63 ± 10 days) via dual-energy x-ray absorptiometry (DXA) scanning. All pigs were genotyped using Illumina Porcine 60k SNP Beadchip and a combined linkage disequilibrium-linkage analysis was used to identify genome-wide significant associations for collected phenotypes. We identified 229 QTLs which associated with adiposity- and metabolic phenotypes at genome-wide significant levels. Subsequently comparative analyses were performed to identify the extent of overlap between previously identified QTLs in both humans and pigs. The combined analysis of a large number of obesity phenotypes has provided insight in the genetic architecture of the molecular mechanisms underlying these traits indicating that QTLs underlying similar phenotypes are clustered in the genome. Our analyses have further confirmed that genetic heterogeneity is an inherent characteristic of obesity traits most likely caused by segregation or fixation of different variants of the individual components belonging to cellular pathways in different populations. Several important genes previously associated to obesity in human studies, along with novel genes were identified. Altogether, this study provides novel insight that may further the current understanding of the molecular mechanisms underlying human obesity.

## Introduction

Obesity, a condition represented by excessive accumulation of body fat, incurs massive economic costs and predisposes individuals to a number of other diseases including diabetes, cardiovascular disorders and osteoarthritis [1, 2]. Obesity is estimated to increase medical expenses by as much as 2,741 US dollars per person every year [1], and its prevalence is rapidly increasing worldwide. The etiology of obesity is highly complex and influenced by numerous factors including genetics and environmental factors such as diet and exercise. Past studies [3] have demonstrated genetic factors to determine as much as 60–70% of phenotypic variation, though genetic determinants underlying only 10% of the total genetic variance have been identified so far [4]. Genetic heterogeneity, confounding between genetics, epigenetic and environmental factors together with imprecise, costly and difficult measurement systems associated with obesity phenotypes, are some of the factors that are likely to contribute to the discrepancy between the overall genetic contribution to obesity and the identified genetic determinants.

For a complex trait like obesity, animal models can aid and accelerate the identification of underlying genetic determinants. Advantages of animal models include the possibility to design populations with certain genetic characteristics and much better control over environmental factors. Mouse models have been widely used primarily due to their evolutionary proximity to humans, their well characterized genome and the relatively low costs involved in housing, handling and breeding them in controlled environments. However, findings from murine models of obesity have often failed to translate to humans largely due to pathophysiological differences [5]. Given these differences, alternative animal models for human obesity are needed where research findings have a greater probability of being translatable to humans. Pig models are of interest in this regard as the pig genome has been sequenced and they are genetically closer to humans especially in the context of energy metabolism and obesity [6, 7]. Pigs are omnivores like humans, and unlike mice, also exhibit almost all of the pathophysiological features related to obesity and metabolic syndrome in a relatively short time span [7].

Given the potential benefits of using pigs to model human obesity, comprehensively phenotyped and genotyped porcine F2 intercross populations were established as a resource for obesity studies. Genetic determinants (Quantitative Trait Loci–QTLs) underlying a broad range of obesity and metabolic phenotypes were identified via combined linkage disequilibrium linkage analysis (LDLA). Subsequently, human chromosomal regions syntenic to identified QTL regions were investigated for previously reported associations with phenotypes comparable to those in pigs. Brief descriptions of the resource population and statistical methods are presented herein together with an overview of results from analyses.

## Results and Discussion

**Fig 1:**
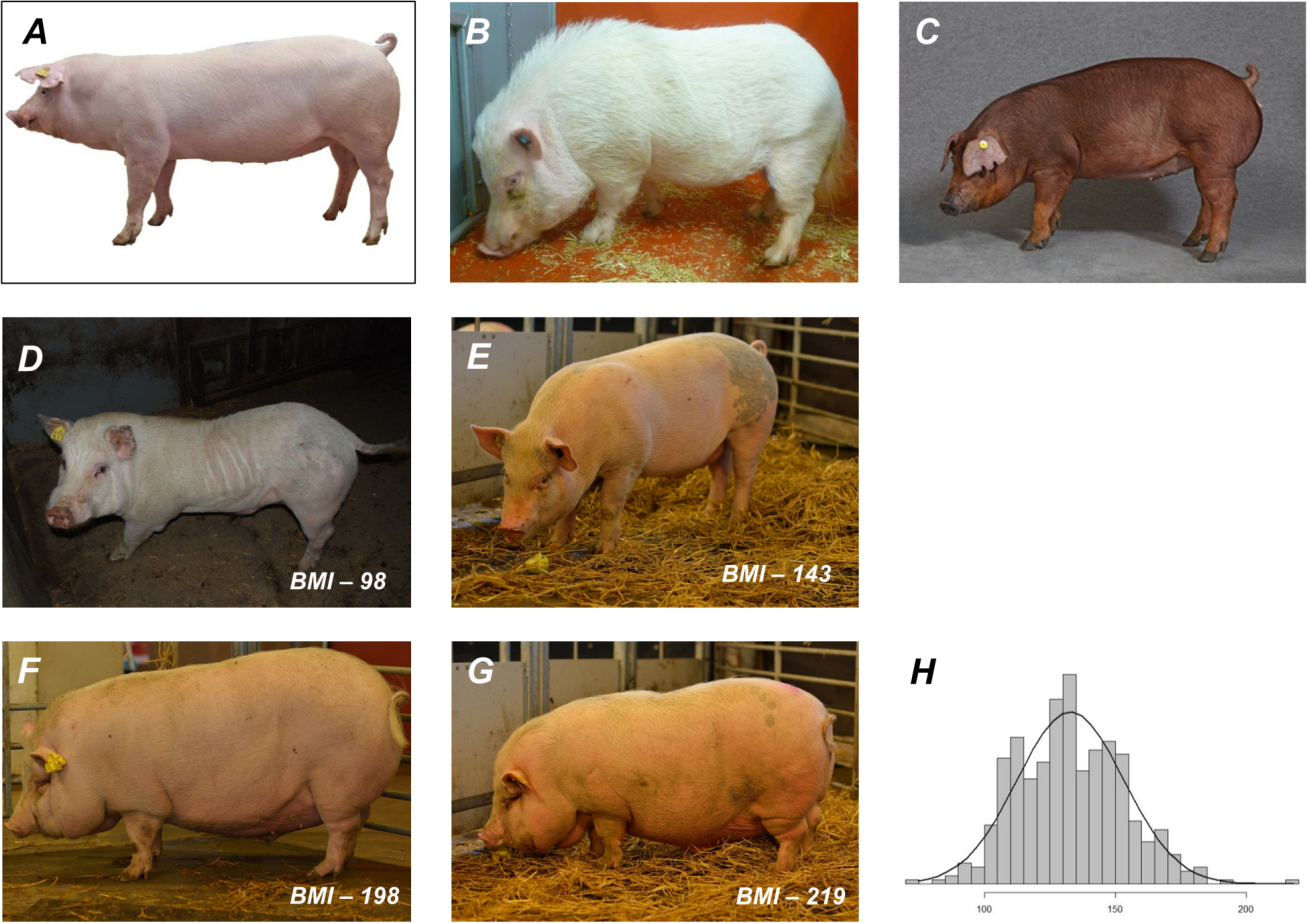
Genetic variance of BMI in F2 population. (A) Yorkshire (B) Minipig (C) Duroc are representative images of pig breeds used to create the F2 resource population. D-G are representative images of animals from the F2 population. H represents the distribution of BMI in F2 pigs measured at 220 ± 45 days. (Photos: A and C courtesy DanBred; B courtesy Ellegaard Göttingen Minipigs; D, E, F, G courtesy Thomas Jakob Olsen).

The overall aim of this study was to identify genetic determinants underlying a broad range of obesity phenotypes in a porcine resource population, and also to evaluate the efficacy of using a porcine model of human obesity for genomic investigations. The porcine resource population was constructed by crossing two sets of Göttingen minipig boars with Duroc and Yorkshire sows separately. Göttingen minipigs are susceptible to diet-induced obesity, and by crossing them to commercial pig breeds that have been genetically selected for leanness over several generations, we aimed to maximize genetic variance for obesity phenotypes in the resultant F2 populations (see Fig 1 for example). The pigs used in this study were raised in highly controlled conditions in order to minimize variation associated with environmental factors, and were subsequently extensively phenotyped. Consequently, these phenotypes (e.g. the body adiposity index, BAI and body mass index, BMI) more accurately represent genetic variation as opposed to corresponding human phenotypes that also include substantial environmental variation.

In order to perform genome-wide association analyses, a strategy based on combined LDLA was used instead of traditional single marker analyses. Given that the resource population was based on an F2 design with a structured pedigree, combined LDLA offered the opportunity to leverage linkage disequilibrium (LD) both within families and across the population, thereby mapping QTLs with narrower confidence intervals [8]. This is in contrast to traditional single marker GWAS which only leverages population-wide LD. On the other hand, since extensive LD has been demonstrated in several livestock genomes, it should be kept in mind that combined LDLA may not offer significant advantage in terms of resolution compared to GWAS in genomic regions of high LD. By mapping QTLs separately in the two crosses derived from Durocs and Yorkshires, we hoped, to be able to exploit differences in the breed-specific LD structure to map QTLs segregating in both crosses to narrower chromosomal regions. This however, could not be exploited to a great extent since few QTLs underlying obesity and metabolic traits of comparative interest were found to segregate in both crosses. This may be due to limitations associated with statistical power and sample size that did not allow the identification of all QTLs in both crosses, or due to founder effects associated with the limited number of animal used in the founding generation. Although, compared to the human population the individual pig breeds are much more homogeneous genetically, the fact that different QTLs segregate in the two breeds supports the hypothesis that genetic heterogeneity is inherent to obesity traits. Combined LDLA is based on a linkage disequilibrium multi-locus iterative peeling (LDMIP) algorithm [8] that uses marker information surrounding a locus to compute IBD probabilities. Since the pig genome is incompletely annotated and some markers are either misplaced or their position is not currently known, this could potentially influence the analyses and should be considered as a potential limitation.

A total of 229 QTLs for 35 different phenotypes (Table 1) were identified as genome-wide significant (S1 Table). Some of these overlap with QTLs for comparable phenotypes in human syntenic regions. Overlapping QTLs for comparable phenotypes in human syntenic regions are indicative of similar genetic mechanisms driving obesity phenotypes in both pigs and humans. Therefore, it was important for us to assess the extent of overlap to determine the efficacy of using pigs as a model for human obesity. However, a few limitations were associated with our assessment of this overlap. Firstly, the NHGRI GWAS catalog [9] was used as a reference database for identification of comparable QTLs in human syntenic regions and our analysis was confined to results included in this catalog. Secondly, syntenic human chromosomal regions could not be defined for all porcine QTLs. This was primarily due to QTLs spanning synteny breakpoints and to ambiguities in the assembly of the porcine genome.

**Table 1:** Description of phenotypes measured in pigs at different ages, and covariates used in the statistical model for association analyses.

*Obesity Phenotypes*		*Model Covariates*
***back_fat1***	Thickness of Subcutaneous Adipose Tissue in Lower Trunk (Measured in mm at Age 3)	Sex, Age 3, (Age 3)^2^
***back_fat2***	Thickness of Subcutaneous Adipose Tissue in Upper Trunk (Measured in mm at Age 3)	Sex, Age 3, (Age 3)^2^
***bai_g***	Body Adiposity Index (Measured at Age 2)	Sex, Age 2, (Age 2)^2^
***bai_s***	Body Adiposity Index (Measured at Age 1)	Sex, Age 1
***birth_wgt***	Birth Weight (Measured in Kgs)	Sex
***bmi_g***	Body Mass Index (Measured at Age 2)	Sex, Age 2, (Age 2)^2^
***bmi_s***	Body Mass Index (Measured at Age 1)	Sex, Age 1
***dg1***	Average Daily Weight Gain from Birth to Age 1 (Weight in Kgs)	Sex, Age 1
***dg2***	Average Daily Weight Gain From Age 1 to Age 2 (Weight in Kgs)	Sex, Age 2, (Age 2)^2^
***mes_fat***	Excision of an 8 cm Diameter Section of Mesenteric Fat in the Triangle Between Ileum and Cecum (Weight in gms)	Sex, Age 3, (Age 3)^2^
***leaf_fat***	Blunt Removal of Retroperitoneal Fat (Weight in Kgs)	Sex, Age 3, (Age 3)^2^, Length (Age 3)
***ome_fat***	Blunt Removal of Greater Omentum (Weight in gms)	Sex, Age 3, (Age 3)^2^, Length (Age 3)
***tr_pfat***	Fat Percentage Trunk Region (DXA scanning)	Sex, Age 1
***wb_lean***	Total Lean Mass in Whole Body (DXA scanning, Weight in Kgs)	Sex, Age 1, Length (Age 1)
***wb_pfat***	Fat Percentage in Whole Body (DXA scanning)	Sex, Age 1
***wb_tf***	Total Fat in Whole Body (DXA scanning, Weight in Kgs)	Sex, Age 1, Length (Age 1)
***Blood Glucose and Lipoprotein Phenotypes Measured in Plasma***		
***cetp_per***	Cholesteryl ester transfer protein Activity (CETP activity—Expressed in Percentage at Age 1)	Sex, Age 1
***ce_s***	Esterified Cholesterol (Expressed in mmol/L at Age 1)	Sex, Age 1
***cl_s***	Free Cholesterol (Expressed in mmol/L at Age 1)	Sex, Age 1
***ct_g***	Total Cholesterol (Expressed in mmol/L at Age 3)	Sex, Age 3, (Age 3)^2^
***ct_s***	Total Cholesterol (Expressed in mmol/L at Age 1)	Sex, Age 1
***hdl_c_g***	High-density-lipoprotein Cholesterol (Expressed in mmol/L at Age 3)	Sex, Age 3, (Age 3)^2^
***hdl_c_s***	High-density-lipoprotein Cholesterol (Expressed in mmol/L at Age 1)	Sex, Age 1
***ldl_c_g***	Low-density-lipoprotein Cholesterol (Expressed in mmol/L at Age 3)	Sex, Age 3, (Age 3)^2^
***ldl_c_s***	Low-density-lipoprotein Cholesterol (Expressed in mmol/L at Age 1)	Sex, Age 1
***pl_s***	Phospholipids (Expressed in mmol/L at Age 1)	Sex, Age 1
***tg_s***	Triglycerides (Expressed in mmol/L at Age 1)	Sex, Age 1
***fructosamin***	Fructosamin (Expressed in μmol/L at Age 3)	Sex, Age 3, (Age 3)^2^
***glucose***	Fasting Glucose (Expressed in mmol/L at Age 3)	Sex, Age 3, (Age 3)^2^
***lipase***	Lipase (Expressed in U/L at Age 3)	Sex, Age 3, (Age 3)^2^
***Blood Glucose and Lipoprotein Phenotypes Measured in ApoB depleted Plasma***		
***hdl_ce_s***	High-Density-Lipoprotein Esterified Cholesterol (Expressed in mmol/L at Age 1)	Sex, Age 1
***hdl_cl_s***	High-Density-Lipoprotein Free Cholesterol (Expressed in mmol/L at Age 1)	Sex, Age 1
***hdl_ct_s***	High-Density-Lipoprotein Cholesterol (Expressed in mmol/L at Age 1)	Sex, Age 1
***hdl_pl_s***	High-Density-Lipoprotein Phospholipids (Expressed in mmol/L at Age 1)	Sex, Age 1
***hdl_tg_s***	High-Density-Lipoprotein Triglycerides (Expressed in mmol/L at Age 1)	Sex, Age 1

*Age 1*: *63 ± 10 days; Age 2*: *218 ± 45 days; Age 3*: *242 ± 48 days*

In addition to overlapping human QTLs, several overlapping pig QTLs for comparable or related phenotypes (e.g. subcutaneous fat, BMI, BAI etc.) were also identified by querying the AnimalQTLdb [10] (S1 Table). Contrary to the NHGRI GWAS catalog, the AnimalQTLdb does not use predefined criteria to determine inclusion of QTLs, but instead exhaustively curates all previously reported QTLs in the literature. Several of these QTLs have confidence intervals that span across the length of entire chromosomes. Consequently, AnimalQTLdb was only queried for previously reported pig QTLs up to 3 Mb in size that overlapped QTLs identified in this study. Thus, information on potential overlap between QTLs spanning larger regions has not been included.

**Fig 2:**
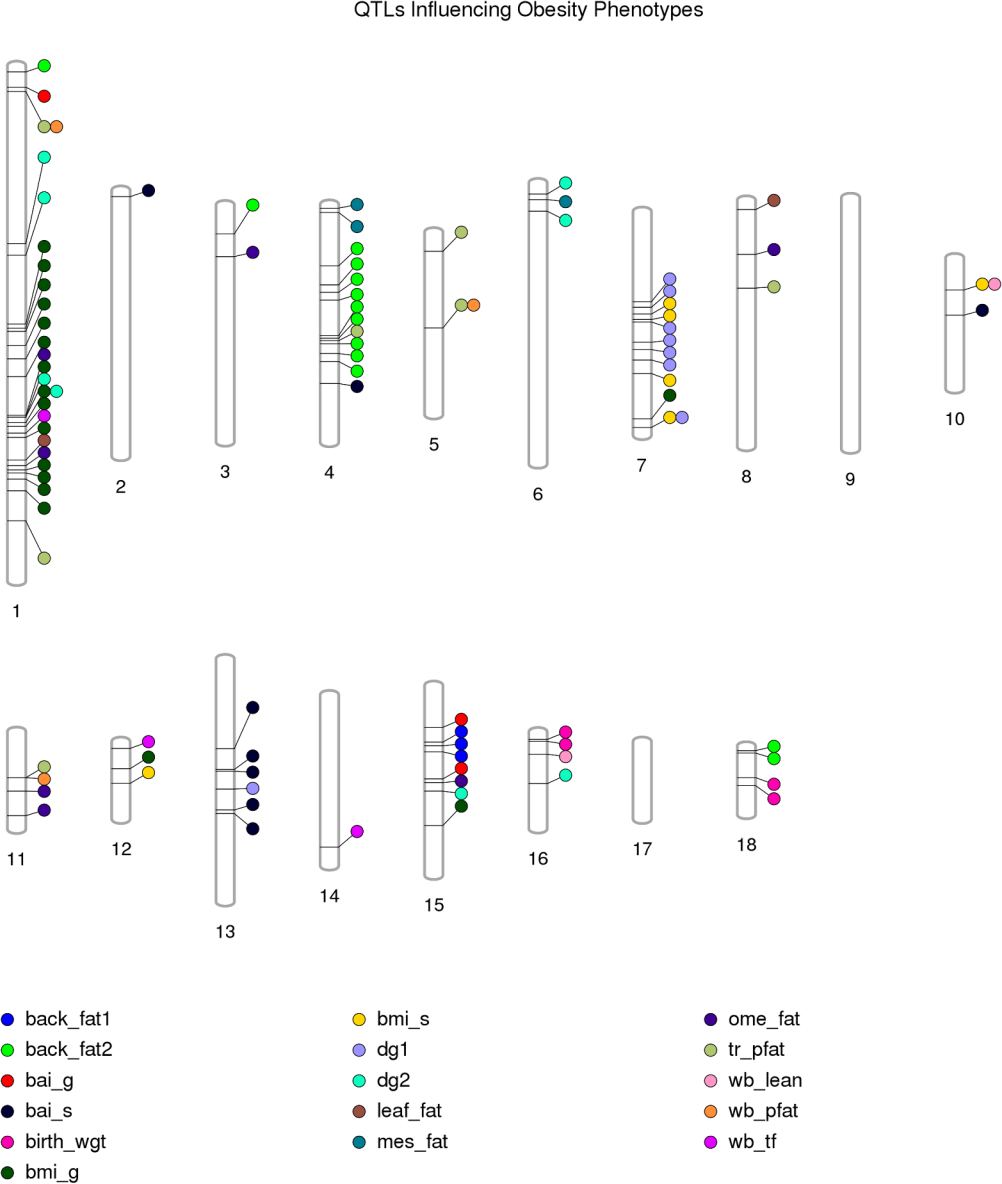
QTLs influencing Obesity related Phenotypes in pigs. Fig 2. (Figure created using Phenogram- http://visualization.ritchielab.psu.edu/phenograms/plot). Vertical columns labeled 1–18 represent porcine autosomes SSC1–18. QTL locations are marked on the chromosomes using a proximity algorithm that minimizes the overlap between individual QTLs for different phenotypes. Different phenotypes are represented by circles filled with different colors. The description of the abbreviated phenotypes is presented in Table 1.

**Fig 3:**
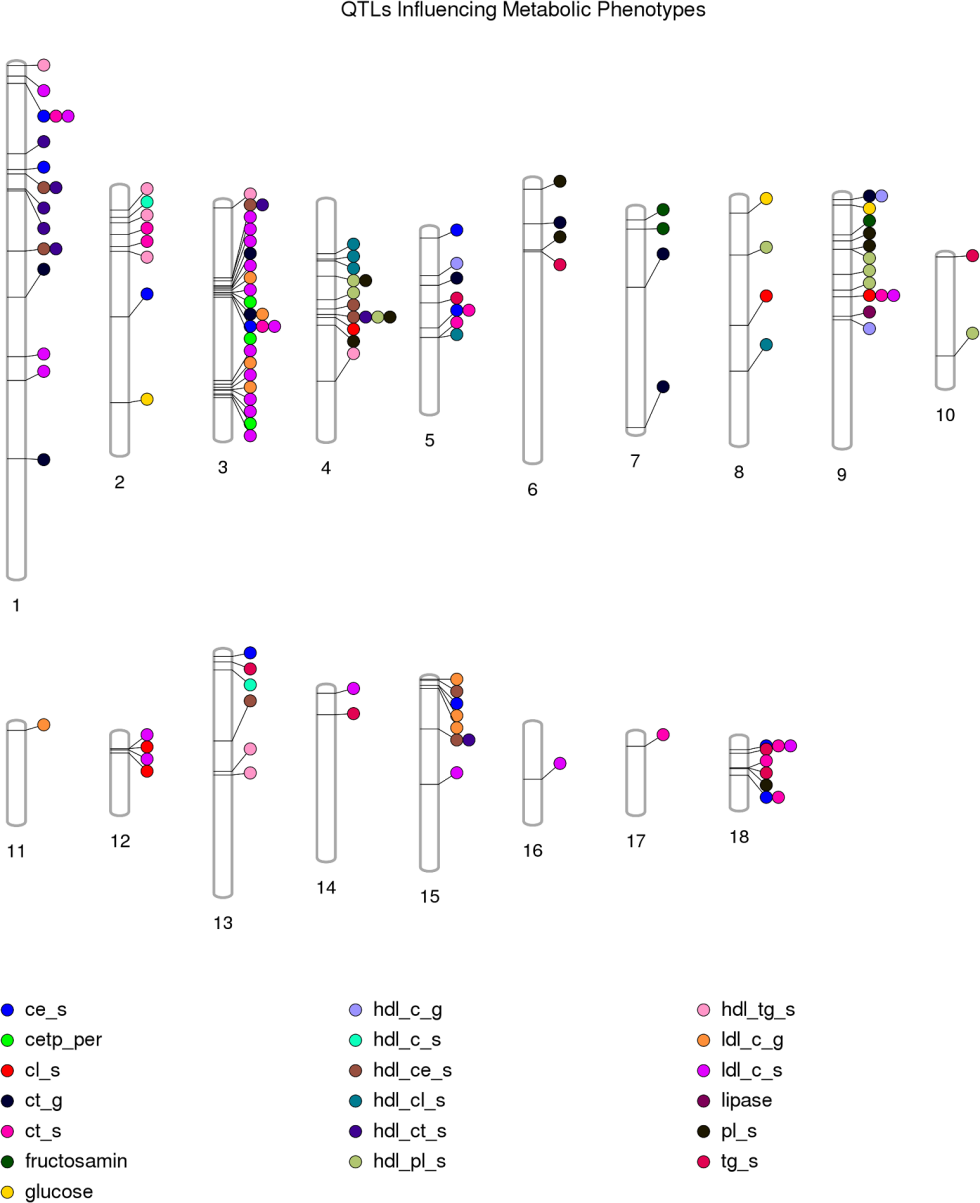
QTLs influencing Metabolic Phenotypes in pigs. Fig 3. (Figure created using Phenogram- http://visualization.ritchielab.psu.edu/phenograms/plot). Vertical columns labeled 1–18 represent porcine autosomes SSC1–18. QTL locations are marked on the chromosomes using a proximity algorithm that minimizes the overlap between individual QTLs for different phenotypes. Different phenotypes are represented by circles filled with different colors and the description of the abbreviated phenotypes is presented in Table 1.

Of the 35 porcine phenotypes analyzed in this study, 11 phenotypes constituted 114 QTLs, of which 20 had QTLs for comparable or related phenotypes in human syntenic regions (Table 2)**.** All porcine autosomes had at least three QTLs each, with the exception of chromosome 17 that harbored a single QTL for total cholesterol measured directly in plasma (*ct_s*). Porcine autosome 1 (*SSC1*) harbored the maximum number (n = 44) of QTLs that represented 16 out of the 35 different phenotypes analyzed in this study. However, the highest density of QTL is on *SSC13*, harboring 0.83 QTLs per Mb (Figs 2 and 3). Overall, clustering of QTLs underlying similar phenotypes can be observed providing support for the notion that genes assigned to the same pathway are clustered in the genome [11].

**Table 2:** Porcine QTLs with overlapping QTLs for related phenotypes in the NHGRI GWAS catalog.

*Pig QTL Data*					*Position of Strongest Association*		*Human Syntenic Region*			*Association Data from NHGRI GWAS Catalog*					
*Pig Trait*	*QTL no*.	*Chr*	*start*	*end*	*Position*	*P-value*	*Chr*	*start*	*end*	*Disease/Trait*	*SNP*	*Position*	*Context*	*P-value*	*PubMed ID*
***back_fat2***	***1***	4	30776117	46626364	37649495	1.91E-06	8	90948835	106785287	Fat distribution (upper trunk subcutaneous adipose tissue)	rs921231	91348168	intron	1.00E-06	21897333
										Fat distribution (upper trunk)	rs921231	91348168	intron	2.00E-06	21897333
										Type 2 diabetes	rs7845219	94925274	Intergenic	6.00E-08	24509480
										Fat distribution (HIV) (arm)	rs10504906	91406400	Intergenic	8.00E-06	21897333
										Type 2 diabetes	rs896854	94948283	intron	1.00E-09	20581827
	***2***	4	54865387	75907976	58665823	1.05E-07	8	59931695	83944860	Obesity	rs4735692	75703428	Intergenic	4.00E-10	23563607
										Body mass index	rs2922763	75661476	Intergenic	6.00E-08	20935630
										Waist circumference	rs4471028	74382740	Intergenic	2.00E-07	17903300
										Visceral fat (overall)	rs16909318	81532989	intron	7.00E-07	22589738
	***3***	4	90666702	91379468	91379468	7.25E-05	1	162832505	163588483	Response to mTOR inhibitor (rapamycin)	rs2063142	163083499	Intergenic	4.00E-06	24009623
***bai_s***	***4***	13	135407662	137685786	135827193	1.58E-05	21	25949345	30230851	Visceral fat (men)	rs17744121	29341277	intron	6.00E-06	22589738
										Body mass index	rs933117	29728478	intron	6.00E-06	22446040
										Response to mTOR inhibitor (everolimus)	rs2832270	29222211	intron	8.00E-06	24009623
										Response to statin therapy (Triglyceride, sum)	rs9305406	30013925	Intergenic	8.00E-06	20339536
***bmi_g***	***5***	1	168007706	172796697	171186575	3.32E-05	15	65037305	67656720	Subcutaneous adipose tissue (overall)	rs11858577	66774225	intron	9.00E-06	22589738
	***6***	1	187060595	195320076	190184135	1.20E-05	14	48827633	57582560	Body mass index (interaction)	rs7350721	55866795	Intergenic	6.00E-07	23192594
										Visceral adipose tissue/subcutaneous adipose tissue ratio (women)	rs8013477	49067707	Intergenic	4.00E-06	22589738
										Visceral fat (men)	rs1530947	49484632	Intergenic	5.00E-06	22589738
	***7***	1	196439311	215457206	214926348	3.67E-06	9	16239003	27329259	Body mass index (asthmatics)	rs3780215	19579429	intron	7.00E-06	23517042
										Obesity-related traits (Fat mass)	rs1340043	18458070	Intergenic	9.00E-06	23251661
										Obesity-related traits (Trunk fat mass)	rs6475216	18444140	Intergenic	9.00E-06	23251661
										Visceral adipose tissue/subcutaneous adipose tissue ratio (overall)	rs4978053	26208859	Intergenic	6.00E-06	22589738
										Fat distribution (HIV) (upper trunk)	rs1944766	18215282	Intergenic	3.00E-06	21897333
										Quantitative traits (Waist Circumference)	rs613391	22670716	intron	5.00E-06	19197348
										Quantitative traits (Weight)	rs2225614	24111282	Intergenic	3.00E-06	19197348
										Type 2 diabetes	rs10811661	22134095	Intergenic	1.00E-27	24509480
														1.00E-18	23945395
														7.00E-07	19056611
														8.00E-15	17463248
														5.00E-08	17463246
														5.00E-06	17463249
										Type 2 diabetes	rs2383208	22132077	Intergenic	2.00E-29	19401414
														3.00E-17	22961080
														3.00E-06	23209189
										Type 2 diabetes	rs10965250	22133285	Intergenic	1.00E-10	20581827
										Type 2 diabetes	rs7020996	22129580	Intergenic	2.00E-07	18372903
										Type 2 diabetes	rs7018475	22137686	Intergenic	3.00E-08	22293688
										Type 2 diabetes	rs1333051	22136490	Intergenic	6.00E-10	21573907
										Type 2 diabetes	rs564398	22029548	ncRNA	1.00E-06	17463249
	***8***	1	220435225	222956776	220558073	1.56E-05	9	8638449	10943443	Obesity-related traits (Dinner intake, adj TEE)	rs294845	10139580	intron	7.00E-06	23251661
										Type 2 diabetes	rs649891	10430602	intron	6.00E-06	21647700
										Type 2 diabetes	rs17584499	8879118	intron	9.00E-10	20174558
	***9***	1	227146232	229129499	227628153	6.60E-06	9	2865933	4911598	Type 2 diabetes	rs7041847	4287466	intron	5.00E-06	24509480
														2.00E-14	22158537
										Type 2 diabetes	rs10814916	4293150	intron	6.00E-12	22961080
	***10***	1	246441732	247595657	247234467	1.03E-05	9	34718652	35787536	Weight (males)	rs10972341	35141708	Intergenic	9.00E-06	19851299
	***11***	1	248776413	251138962	249132600	1.64E-05	9	36738650	38472147	Obesity and blood pressure (BMI)	rs16933812	36969208	intron	5.00E-06	22013104
										Obesity and blood pressure (Total Fat Mass)	rs16933812	36969208	intron	9.00E-09	22013104
***ce_s***	***12***	3	56636950	57391731	56723081	2.92E-06	2	81091886	81826661	Bilirubin levels	rs12052359	81645798	Intergenic	7.00E-06	22085899
***dg1***	***13***	7	60925144	76495215	67628895	1.27E-07	14	29020842	39120983	Body mass index	rs11847697	30045906	Intergenic	2.00E-06	23669352
										Thyroid hormone levels (TSH)	rs1537424	36104812	intron	1.00E-08	23408906
										Obesity-related traits (Diet Fat)	rs718545	30020417	Intergenic	9.00E-06	23251661
										Body mass index	rs11847697	30045906	Intergenic	6.00E-11	20935630
	***14***	7	86871288	98757230	90901391	1.37E-06	15	85286426	98423740	Response to mTOR inhibitor (rapamycin)	rs17664713	94775357	Intergenic	5.00E-06	24009623
										Thyroid hormone levels (TSH)	rs17776563	88575873	Intergenic	3.00E-10	23408906
										Fat distribution (HIV) (arm)	rs1993976	98063084	Intergenic	8.00E-07	21897333
										Obesity (extreme)	rs970843	98332800	Intergenic	5.00E-06	21935397
***hdl_ce_s***	***15***	1	66931623	67106130	67106130	7.95E-05	6	96545315	96733074	Coronary heart disease	rs12200560	96632322	Intergenic	6.00E-07	22319020
***hdl_cl_s***	***16***	4	29131900	31654951	31569807	3.03E-06	8	106022233	108323587	Obesity-related traits (HDL)	rs7004587	107028124	Intergenic	3.00E-06	23251661
***hdl_ct_s***	***17***	1	66931623	67106130	67106130	0.000186	6	96545315	96733074	Coronary heart disease	rs12200560	96632322	Intergenic	6.00E-07	22319020
***hdl_tg_s***	***18***	2	38562295	39437513	38837764	2.68E-06	11	17003981	17858922	Type 2 diabetes	rs5215	17387083	missense	3.00E-11	24509480
														4.00E-07	18372903
														5.00E-11	17463249
										Type 2 diabetes (obese)	rs5219	17388025	missense	5.00E-07	19056611
										Type 2 diabetes (non-obese)	rs5219	17388025	missense	1.00E-09	19056611
										Type 2 diabetes	rs5219	17388025	missense	1.00E-07	17463246
														7.00E-11	17463248
***ldl_c_s***	***19***	3	120245799	121054453	120245799	2.78E-05	2	2043854	4034691	Type 2 diabetes	rs11677370	3793830	intron	3.00E-06	21490949
***tg_s***	***20***	6	43445975	43627267	43627267	0.000331	1	3084050	3542414	Response to statin therapy	rs6658356	3363689	intron	2.00E-06	20339536

A substantial proportion of QTLs were identified within the Minipig-Duroc cross compared to the Minipig-Yorkshire cross (S1 Table, second column). We have defined individual significant positions as independent QTLs if located more than 1 cM apart. However, many of the Minipig-Duroc QTLs are located close together and at the same time, extent of LD is greater in the Minipig-Duroc cross (S1 Fig). Hence, our definition of QTLs, while appropriate in the Minipig-Yorkshire cross, may inflate the number of QTLs in the Minipig-Duroc cross by splitting up a single QTL into multiple adjacent QTLs. Also, it should be noted that some of the Minipig-Duroc QTLs are in fact detected in the Minipig-Yorkshire cross, however, at significance levels that are borderline to the level considered to be genome-wide significant in this study (data not shown).

There were 19 chromosomal regions that were associated with more than one phenotype (Fig 2) (Table 3), of which 2 QTL regions on chromosome 3 (*SSC3*:56,689,989 and *SSC3*:56,723,081), and another 2 QTL regions on chromosome 5 (*SSC5*:58,544,792 and *SSC5*:60,364,446) are in so close proximity that they may represent two single QTLs influencing multiple phenotypes. Most of these QTLs (n = 14) influenced cholesterol related phenotypes. Two QTLs on *SSC1* (15,748,172 bp) and *SSC5* (58,544,792 bp) influenced fat percentage of the trunk region as well as of the whole body measured via DXA scanning. QTLs on *SSC1* (218,448,574 bp) and *SSC7* (132,308,360bp) influenced BMI and the average daily weight gain measured at different ages. Finally, one QTL on *SSC10* (19,668,169) influenced both BMI measured at 63 ± 10 days and whole body lean mass measured via DXA scanning at the same age. Hence in most cases, the multiple phenotypes influenced by the same chromosomal position are interrelated phenotypes most likely influenced by similar cellular pathways.

**Table 3:** Chromosomal positions associated with multiple phenotypes in the pig resource population.

*Position*	*Phenotypes*
Chr1:11134679	*ce_s*, *ct_s*, *ldl_c_s*
Chr1:15748172	*tr_pfat*, *wb_pfat*
Chr1:67106130	*hdl_ce_s*, *hdl_ct_s*
Chr1:114718770	*hdl_ce_s*, *hdl_ct_s*
Chr1:218448574	*bmi_g*, *dg2*
Chr3:46117851	*hdl_ce_s*, *hdl_ct_s*
Chr3:56689989	*ct_g*, *ldl_c_g*
Chr3:56723081	*ce_s*, *ct_s*, *ldl_c_s*
Chr4:45434589	*hdl_pl_s*, *pl_s*
Chr4:69198075	*hdl_ce_s*, *hdl_ct_s*, *hdl_pl_s*, *pl_s*
Chr5:58544792	*tr_pfat*, *wb_pfat*
Chr5:60364446	*ce_s*, *ct_s*
Chr7:132308360	*bmi_s*, *dg1*
Chr9:2418857	*ct_g*, *hdl_c_g*
Chr9:62416702	*cl_s*, *ct_s*, *ldl_c_s*
Chr10:19668169	*bmi_s*, *wb_lean*
Chr15:45350470	*hdl_ce_s*, *hdl_ct_s*
Chr18:9431105	*ce_s*, *ct_s*, *ldl_c_s*
Chr18:32618335	*ce_s*, *ct_s*

In addition to identifying QTLs for specific phenotypes, some general inferences can also be drawn with respect to the biology driving different phenotypes. For example, storage of fat in intra-abdominal fat compartments (retroperitoneal fat, mesenteric fat, omental fat) appears to be controlled by separate loci except for a locus from 241.4 to 244.7 Mb on *SSC1* which is associated with both retroperitoneal and omental fat. None of the loci associated with intra-abdominal fat accumulation are associated with accumulation of subcutaneous fat (*back_fat1* and *2*). Even accumulation of subcutaneous fat of upper (*back_fat2*) and lower trunk region (*back_fat1*) seems to be influenced by different genetic loci i.e. lower trunk subcutaneous fat is associated with loci on *SSC15*, whereas upper trunk subcutaneous fat is associated with loci on *SSC1*, *3*, *4* and *18*. A single locus on *SSC1* (15.7 Mb) is associated with both trunk and whole body fat percentage measured via DXA scanning. On the other hand, trunk fat percentage (*tr_pfat*) as measured by DXA scan does not overlap with upper or lower trunk subcutaneous fat except for a locus on *SSC4*, 83 Mb. DXA scanning was performed in the young pig whereas trunk subcutaneous fat was measured in adult pigs. The difference in associated QTL may therefore indicate that different molecular mechanisms are involved in fat deposition in the trunk region at different ages, except for the locus on *SSC4* which seems to have a role independent of age. Another locus on *SSC1* (213.9–215.0 Mb) is associated with weight of omental fat, BMI and daily weight gain in adolescent pigs (*dg2*). Four regions on *SSC3*; around 46.1 Mb, from 51.1 to 58.1 Mb, from 109.5 to 120.2 Mb and from 124.6 to 125.0 Mb, are associated with several blood lipid traits in both early life and during adolescence (Fig 3). Thus, this region seems to harbor a number of different genes affecting different aspects of the phenotypes involved in plasma cholesterol levels indicating that there is genomic clustering of functionally related genes and co-regulatory elements [11]. A locus on *SSC4* (69.2 Mb) is associated with total HDL-cholesterol and esterified HDL-cholesterol but not with overall cholesterol level or LDL-cholesterol levels. The same locus is associated with overall phospholipid level as well as HDL-phospholipid level.

Several QTL regions contained evidence indicative of biological significance. In some cases, similar QTLs have previously been found in human studies but in other cases, the identified QTLs are novel and to our knowledge not described before in humans, rodents or pigs. A selection of the most attractive and biologically significant results is described below:

### Obesity Phenotypes

#### Body Adiposity Index (BAI)

A total of 11 QTLs were identified for BAI, of which 8 QTLs were identified for BAI measured at around two months of age (64 ± 11 days, *bai_s*), and 3 other QTLs were identified for BAI measured at slaughter (220 ± 45 days, *bai_g*). Amongst these, an interesting 2 Mb QTL is located on *SSC13* (135,407,662–137,685,786) that includes two genes *BACH1* and *GRIK1*. The corresponding human syntenic region is located on *HSA21* that also includes both these genes. Human investigations have identified an intronic SNP (rs17744121) in *BACH1* to be associated with visceral fat in men (p = 6.0E-6) [12], and another intronic SNP (rs933117) in *GRIK1* to be associated with BMI (p = 6.0E-6) [13]. Functionally, BACH1 is a transcription factor that interacts with MAFK, and can suppress expression of heme-oxidase 1. *GRIK1* encodes a glutamate receptor that serves as the predominant excitatory neurotransmitter in the mammalian brain.

#### Body Mass Index (BMI)

A total of 23 QTLs were identified for BMI in this study, 6 of which were identified for BMI measured at around two months of age (64 ± 11 days, *bmi_s*), and another 17 were identified for BMI measured at the time of slaughter (220 ± 45 days, *bmi_g*). Several of these QTLs, particularly those identified for *bmi_g*, are located on *SSC1*, and overlap with QTLs for comparable phenotypes in human syntenic chromosomal regions. For example, QTL5 (Table 2) spanning approximately 5 Mb (168,007,706–172,796,697) contains *SMAD6*, and is syntenic to a 2.5 Mb chromosomal region on *HSA15* containing a *SMAD6* intronic variant (rs11858577) associated with subcutaneous fat tissue volume in men and women (p = 9.0E-06) [12].

QTL6 (Table 2) located on *SSC1* (187,060,595–195,320,076) is syntenic to a chromosomal region on *HSA14* that contains three intergenic SNPs associated with BMI (rs7350721, p = 6.0E-07) [14], visceral adipose tissue to subcutaneous adipose tissue ratio in women (rs8013477, p = 4.0E-06) [12], and visceral fat in men (rs1530947, p = 5.0E-06) [12]. The porcine QTL region along with its corresponding human syntenic region both contains the genes *RPS29*, encoding a ribosomal protein, and *PELI2*, encoding a ubiquitin protein ligase family member. None of these have a known biological function that relate directly to obesity.

QTL7 (Table 2) covers approximately 20 Mb (196,439,311–215,457,206) on *SSC1*. This extended QTL probably represents a series of BMI QTLs on *SSC1* which, however, cannot be precisely delimited in the present study. The region corresponds to a human syntenic region extending more than 10 Mb on *HSA9* which is also rich in obesity QTLs and contains several variants associated with a range of obesity phenotypes like BMI in asthmatic adults (rs3780215, p = 7.0E-06) [15], fat mass (rs1340043, 9.0E-06), trunk fat mass (rs6475216, p = 9.0E-06) [16], subcutaneous adipose tissue of upper trunk in HIV infected men treated with antiretroviral therapy (rs1944766, p = 3.0E-06) [17], weight (rs2225614, p = 3.0E-06), waist circumference (rs613391, p = 5.0E-06) [18] and overall volume of subcutaneous and visceral fat (rs4978053, p = 6.0E-06) [12]. However, none of these human genetic variants have syntenic porcine positions that are in close proximity to the porcine QTL position with the strongest association (p = 3.7E-06). In fact, the position of strongest association within QTL7 is closer to another variant (rs1927702) that has been associated with BMI (p = 6.0E-06) [19], but is located marginally outside the chromosomal extent of QTL7. Though there are three genes contained in the human syntenic region (*ADAMTSL1*, *TUSC1* and *BNC2*), none of these seems to have any presently known biological relationship with obesity or any of its related phenotypes.

QTL10 (Table 2) is a narrow QTL (~ 1.15 Mb) extending between 246,441,732–247,595,657 on *SSC1*, syntenic to a chromosomal regions on *HSA9* that includes a variant (rs10972341) associated with ‘weight in males’ (p = 9.0E-06) [19].

QTL11 (Table 2) on SSC1 extending between 248,776,413–251,138,962 includes *PAX5*, and is syntenic to a narrow human chromosomal region on *HSA9* (36,738,650–38,472,147) that contains an intronic variant (rs16933812) of *PAX5* associated with BMI (p = 5.0E-06) and total fat mass (p = 9.0E-06) [20]. *PAX5* is a member of the PAX transcription factor family with a highly conserved DNA binding motif known as the paired box. PAX5 has been described as a B-cell specific transcription factor and its dysregulation is associated with different types of leukemia. The gene is also expressed in brain and testes. The protein plays a role in cell proliferation and is an important regulator in early development.

#### Upper Trunk Subcutaneous Fat

A total of 13 QTLs for Upper Trunk Subcutaneous Fat were identified in this study, of which 3 QTLs located on *SSC4* overlap with QTLs for comparable traits in corresponding human syntenic chromosomal regions.

QTL 1 and 2 (Table 2) are large QTLs and consequently contain several relevant associations reported in human syntenic regions. However, none of them seems to be located close to corresponding porcine chromosomal positions with strongest associations within the QTL regions. For example, QTL1 (*SSC4*:30,776,117–46,626,364) with its strongest association at *SSC4*:37,649,495, is syntenic to *HSA8* (90,948,835–106,785,287) that contains several variants associated with upper trunk subcutaneous adipose tissue (rs921231, *HSA8*:91,348,168) [17], arm fat distribution (rs10504906, *HSA8*:91,406,400) [17], and Type 2 diabetes (rs7845219, *HSA8*:94,925,274; rs896854, *HSA8*:94,948,283) [21, 22]. One of these genetic variants (rs921231) is located in the intron of *SLC26A7* that belongs to a family of anion transporters (solute carrier family) reported to be primarily involved in renal physiology [23]. Other members of the solute carrier family have been implicated in obesity [24].

Similarly QTL2 (*SSC4*:54,865,387–75,907,976) with its strongest association at *SSC4*:58,665,823, is syntenic to *HSA8* (59,931,695–83,944,860) that contains several variants associated with obesity (rs4735692, *HSA8*:75,703,428) [25], BMI (rs2922763, *HSA8*:75,661,476) [4], waist circumference (rs4471028, *HSA8*:74,382,740) [26], and overall visceral fat (rs16909318, *HSA8*:81,532,989) [12]. One variant (rs16909318) is located in the intron of *FABP12* that belongs to a family of fatty acid transport proteins that has been linked to both obesity [27] and metabolic syndrome [28].

### Blood Glucose and Lipoprotein Phenotypes

#### Fasting glucose

Three distinct QTL regions were identified to be significantly associated with fasting glucose levels measured at 242 ± 48 days of birth, of which one QTL position on *SSC9* (5,534,785, p = 3.7E-04) is located within an intron of *STIM1*. This is a novel QTL and association between fasting glucose and this locus has, to our knowledge, not been reported before neither in rodents nor in humans or pigs. Due to its localization within *STIM1* it is however a very interesting QTL. This gene encodes a transmembrane calcium sensor located on the endoplasmic reticulum that regulates store-operated calcium entry (SOCE). In-vitro studies using insulin secreting beta cell lines indicate that non-specific inhibitors of SOCE (e.g. SKF-96365) can inhibit glucose-induced insulin secretion in these cells [29]. Murine studies have demonstrated that high glucose levels can induce *Stim1* expression in micro vessel endothelial cells [30] and can restore coronary endothelial function in type 1 diabetic mice [31].

#### Free Cholesterol

A total of 5 QTLs were identified for free cholesterol measured directly in plasma. One QTL on *SSC12* (12,081,814, p = 4.0E-04) is especially interesting since it has not been identified in rodents or humans before, and because it is located within an intron of *STRADA*. This gene encodes an adaptor protein that interacts and activates STK11 (Also known as LKB1), which in turn phosphorylates and activates AMPK, a central metabolic sensor that regulates lipid, cholesterol and glucose metabolism in liver, muscles and adipose tissue [32].

Free cholesterol was also measured in apoB depleted plasma and 5 QTLs were identified for this phenotype. One QTL (QTL16, Table 2) had a matching QTL for HDL in its corresponding human syntenic region. QTL16 extends between 29,131,900–31,654,951 on *SSC4*, and is syntenic to *HSA6* (96,545,315–96,733,074) containing an intergenic variant (rs7004587) associated with HDL cholesterol (p = 3.0E-06) [16] that is located between three genes *ANGPT1*, *OXR1*, and *ABRA*/*STARS*. Mechanistically, there is no evidence that indicates a role of either of these genes in regulating plasma cholesterol levels. However, *STARS* (striated muscle activator of Rho signaling), encodes a membrane bound protein expressed in cardiac and striated muscles that enhances Rho-dependent transcription in muscle cells [33]. Rho-GTPases are small signaling G proteins that can also be activated by HDL proteins (e.g. ApoA1) to influence ‘reverse cholesterol transport’ via HDL carriage from peripheral tissues to liver for eventual elimination from the body [34]. The colocation of human and porcine QTLs in syntenic chromosomal regions together with its known biological function, make STARS a putative candidate for further studies with respect to cholesterol levels in plasma.

#### Esterified Cholesterol in Plasma

A total of 10 QTLs were identified for this phenotype, of which only one QTL (QTL12, Table 2) had a QTL for an indirectly comparable phenotype in the corresponding human syntenic region. QTL15 extends over a narrow chromosomal region on *SSC3* (56,636,950–57,391,731) and is syntenic to *HSA2*:81,091,886–81,826,661 that contains a genetic variant (rs12052359, *HSA2*:81,645,798) associated with serum bilirubin levels in an African American population (p = 7.4E-06) [35]. While both the porcine QTL region and the corresponding human syntenic regions do not contain any known genes, the colocation of these QTLs is interesting because serum bilirubin concentration is known to be inversely correlated to concentration of esterified cholesterol in serum [36].

#### Low Density Lipoprotein Cholesterol

A total of 22 QTLs (the maximum for any phenotype in this study) were identified for low density lipoprotein cholesterol measured directly in plasma at 63 ± 10 days of birth. However, half of these QTLs (n = 11) are located on *SSC3* in two separate chromosomal regions extending over approximately 10 Mb. One of these QTL positions on *SSC3* (115,804,895, p = 8.2E-06) is a novel QTL not previously reported in rodents or human. It is located in an intron of *LPIN1* encoding a phosphatidic acid phosphohydrolase that is a member of a broader family of Lipin proteins that play key roles in triglyceride and membrane phospholipid biosynthesis [37]. Murine studies indicate that *Lipin* expression can influence fat storage capacity of adipocytes and whole-body energy expenditure and fat utilization, both of which can directly influence obesity [38]. Human studies have also found *LPIN1* expression in visceral adipose tissue to be correlated with body fat percentage, plasma triglyceride level and plasma leptin level. Additionally, *LPIN1* mRNA levels have been found to be positively correlated with *PPARG* and *ADIPOQ* mRNA levels in visceral and subcutaneous adipose tissue [39]. *PPARG* and *ADIPOQ* are highly expressed in adipose tissue [40, 41]; are involved in cholesterol homeostasis, differentiation of adipocytes and accumulation of lipids (PPARG) [42–44]; and in modulation of glucose levels and fatty acid oxidation (ADIPOQ) [45].

#### High Density Lipoprotein Cholesterol

High density lipoprotein-cholesterol measured directly in plasma at 63 ± 10 and 242 ± 48 days of birth; and indirectly at 63 ± 10 days of birth in apoB depleted (high-density-lipoprotein fraction) plasma. All these measurements were treated as separate phenotypes in the analyses and a total of 26 QTLs were identified, of which 5 chromosomal regions are associated with two different measures of cholesterol and therefore represent 10 QTLs. Two additional sets of QTLs are within 1.2 Mb of each other that could represent two QTLs instead of four.

In the context of high-density-lipoprotein cholesterol, an interesting novel QTL associated with both total cholesterol (p = 1.2E-04) and its esterified fraction (p = 2.1E-04) in apoB depleted plasma was identified on *SSC1* (114,718,770). This chromosomal position is located in the intron of the *RORA* gene that encodes a receptor for cholesterol sulphate, 7-dehydroxycholesterol and cholesterol [46–48]. Functionally, it is a key regulator of cholesterol levels [49]. We categorize this QTL as a novel QTL even though human studies have identified genetic variants around *RORA* that are weakly associated with cholesterol (both HDL and LDL fractions) [50]

### Conclusion

The combined analysis of a large number of obesity phenotypes has provided new and confirmed previous insight in the genetic architecture of the molecular mechanisms underlying these traits. Our analyses have further confirmed that genetic heterogeneity is an inherent characteristic of obesity traits most likely caused by segregation or fixation of different variants of the individual components belonging to cellular pathways in different populations. Overall, several QTLs reported in this study are in good accordance with previously reported QTLs for comparable or related phenotypes in pigs (S1 Table). Several of these QTLs also overlap with previously reported QTLs for comparable human phenotypes which indicate that similar genetic mechanisms drive obesity phenotypes in both pigs and humans. The study provides support for novel QTL regions and candidate genes for obesity and metabolic traits which can be exploited in future whole genome sequencing projects in humans. Several possibilities of further analyses of causative variants and molecular pathways exist since the porcine resource described in this study has not only been extensively phenotyped and genotyped, but also subjected to extensive tissue sampling at slaughter. Results of such future investigations could provide valuable and novel biological insights into obesity that could potentially be translatable to humans.

## Materials and Methods

### Experimental Design and Genotyping

The resource population was established in the following way: In the parental generation seven purebred Yorkshire (YY) sows and seven purebred Duroc (DD) sows from a DanBred breeding herd were mated to 14 Göttingen Minipig (MM) boars from Ellegaard A/S (all animals unrelated at the grandparental level). Among the DM F1 animals 28 gilts and 16 boars were mated to produce 285 animals; among the YM F1 animals 26 gilts and 13 boars were mated to produce 279 animals. The animals were produced and slaughtered in three batches with approximately the same number of F2 animals from the Duroc and Yorkshire crosses in each batch. The pigs were kept under normal condition for production pigs in Denmark in pens with 10–15 animals per pen at a temperature around 20±3°C with ad libitum administration of standard pig feed and free access to water. Both Duroc and Yorkshire are production breeds that have undergone extensive selection for leanness and growth traits, while Göttingen minipigs are mainly used for research purposes and are bred primarily for their small size and ease of handling. Unlike the production pigs, Göttingen minipigs are also susceptible to diet induced obesity and share many metabolic dysfunctions associated with human obesity [51] (Fig 1). All 564 pigs were genotyped using Illumina Porcine 60k SNP Beadchip.

The project was approved by the Danish Animal Experimentation Board. Animal care and maintenance have been conducted according to the Danish “Animal Maintenance Act” (Act 432 dated 09/06/2004). The animals were housed at a regular pig farm, and slaughtered at a commercial slaughterhouse by stunning and bleeding under veterinary supervision. Tissue and blood samples were collected at slaughter.

### Collection of Phenotypes

Extensive phenotypic collection was performed from birth to slaughter (242 ± 48 days) including obesity, obesity-related, and metabolic phenotypes; and measurements of fat compartments at slaughter. In addition, body composition was determined after weaning using dual-energy x-ray absorptiometry (DXA) scanning at about two months of age (63 ± 10 days). Further details of pedigree and phenotyping of obesity traits are available in Kogelman et al. [52]. Plasma lipid levels were assayed by standardized techniques using a Konelab 20 Clinical Chemistry Analyzer (Thermo Scientific, Sweden) and commercial reagent kits from Roche Diagnostics for Total Cholesterol (CT) and from ThermoElectron for triglycerides (TG) and High Density Lipoprotein Cholesterol (HDL-C) levels (direct method). Free cholesterol (CL) and phospholipid concentrations were measured using reagents from Diasys, Germany. Cholesteryl ester (CE) mass was calculated as CT–CL. Fasting plasma Low Density Lipoprotein-Cholesterol (LDL-C) was calculated using the Friedewald formula [53]. Plasma HDL-C levels were determined after dextran sulfate-magnesium precipitation of apolipoprotein B-containing lipoproteins. Plasma CETP activity was assayed by using the method of Guerin et al. [54], which estimates CE transfer from HDL to apoB-containing lipoprotein particles (expressed as percentage). A list of the 35 phenotypes included in the study is provided in Table 1.

### Statistical Analyses

Phenotype data were checked for normality and log or square-root transformations were applied when required. Four phenotypes had 1–3 data points that were several standard deviations (5–13) away from the mean, and were consequently considered outliers that were excluded prior to analyses. Statistical analyses were carried out separately within the Duroc and Yorkshire crosses. Preliminary quality control of genotype data was performed by excluding all SNPs that had a minor allele frequency (MAF) < 0.05, Hardy Weinberg equilibrium test p-value < 0.001, and a genotype call rate < 0.95.

Subsequently, identity by descent (IBD) probabilities were estimated chromosome-wise for each sliding marker bracket at its midpoint using the linkage disequilibrium (LD) multi-locus iterative peeling (LDMIP) method as described by Meuwissen and Goddard [8]. Variance component analysis was then performed with ASReml [55] using a mixed linear model. Genome-wide association analysis was performed via a likelihood ratio test, where the test statistic was calculated as follows:

$$2\Delta l=2\left(l_{q}\;-\;l_{n}\right)\;≅\;\chi^{2}\;with\;1\;d.f.$$

Where:

***- 2Δl*** is the likelihood ratio test statistic;
***- l***_***q***_ is the maximum likelihood estimate of a full model that included the fixed effect of gender, a number of covariates depending upon the phenotype (Specified in Table 1), a random QTL effect based on the estimated IBD relationships, as well as a numerator relationship matrix to account for polygenic effects. Batch effect due to production of the animals in three contemporary groups was found to be non-significant, and hence excluded as a covariate from statistical analyses. Using matrix notation, the full model can be described as follows:

$$y\;=1\mu +\mathrm{Xb}+Z_{1}u+Z_{2}v+e$$

***y*** = vector of phenotypes
***μ*** = mean
***X***, ***Z***_1_ and ***Z***_2_ are design matrices
***b*** = vector of fixed effects
***u*** = vector of random polygenic effects
***v*** = vector of random QTL effect
***e*** = vector of random residuals.

Assuming the following mutually independent distributions of random variables:

$$u∼N(0,A\sigma_{u}^{2})$$

$$v∼N(0,G\sigma_{v}^{2})$$

$$e∼N(0,I\sigma_{e}^{2})$$

Where: **A** = Additive genetic relationship matrix

**G** = Average Identity by Descent matrix
**I** = Identity matrix
***- l***_***n***_ is the maximum likelihood estimate of a null hypothesis that included all effects in the full model except for the QTL effect.

Level of significance (p-values) was computed by assuming ***2Δl*** to follow a chi-squared distribution with one degree of freedom under the null-hypothesis of no QTL in the tested marker bracket. QTLs with a statistical significance of p<0.0001, or those with a point-wise p<0.001 and whose–log10(p) was >3 times greater than the average–log10(p) in the flanking 5 Mb (i.e. 10 Mbs in total) chromosomal window, were considered to be genome-wide significant. Adjacent significant positions were regarded as individual QTLs if located more than 1 Mb apart.

To evaluate extend of LD in each cross, decay of r^2^ over distance was calculated using the method described by Badke et al. (2012) [56].

### Comparative Analyses

The liftOver tool available via UCSC Genome Browser [57] was used to convert genome coordinates between porcine and human assemblies and to map human chromosomal regions syntenic to porcine chromosomal regions containing QTLs associated with different phenotypes. Since liftovers are currently not available between Sscrofa 10.2 build and the current human genome build, we used Sccrofa 9.2 build for the liftover procedure. Additional information on porcine gene annotation was obtained from Sscrofa 10.2. Successive QTL positions that were genome-wide significant were considered to represent a single QTL whose extent was determined by the genomic positions of the first and the last genome-wide significant QTL position. Chromosomal extents of QTLs smaller than 100 kilobases were extended to a minimum of 100 kilobases. The National Human Genome Research Institute (NHGRI) catalog [9] was used to identify human SNP-trait associations in chromosomal regions syntenic to porcine QTLs. These associations were manually curated to identify SNP associations with human phenotypes that were comparable to the porcine phenotypes. Data on 12,618 pig QTLs from 461 publication representing 656 different traits were also downloaded from the Animal QTL database (Animal QTLdb) [10] and subsequently used to identify previously identified porcine QTLs up to 3 Mb in size that overlapped QTL regions identified in this study. These overlapping QTLs were manually curated to identify phenotypes comparable or related to those identified in the present study. Results of these comparisons are presented in S1 Table. All porcine chromosomal locations described in this study are based on the Sscrofa 9.2 assembly of the pig genome.

## Supporting Information

S1 Fig Decay of average r^2^ over distance. Decay of average r^2^ over distance calculated by the method describe by Badke et al. (2012) [56]. Average LD over short distances corresponds well to within-population LD observed previously [56]. Over longer distances, significantly stronger LD is found in the present cross which is in accordance with the LD generated by crossing different breeds.
(PDF)

S1 Table Complete list of QTLs. Description of the number of QTLs identified for each phenotype, the cross in which they were identified, their genomic position, size and significance levels; along with previously reported QTLs derived from the AnimalQTLdb for comparable phenotypes that overlap QTLs identified in the current study. Name (No. of QTLs) = abbreviations for each phenotype as described in Table 1, along with the number of QTLs identified for this phenotype; Cross = cross in which QTL was identified; Chr = Chromosome; Pos = Chromosomal positions with peak significance (Sscrofa 9.2); start, end = QTL start and end chromosomal positions (Sscrofa 9.2); Pvalue = peak significance of the QTL; Animal_QTL_DB_qtls (QTL id) = Previously reported QTLs for comparable phenotypes derived from the AnimalQTLdb along with their QTL ids.
(XLSX)
