# Supplementary figures and images for: Comparative Analyses of QTLs Influencing Obesity and Metabolic Phenotypes in Pigs and Humans
Source: PLoS One. 2015 Sep 8;10(9):e0137356. doi: 10.1371/journal.pone.0137356 (PMC4562524; doi:10.1371/journal.pone.0137356)

Decay of average  $r^2$  over distance

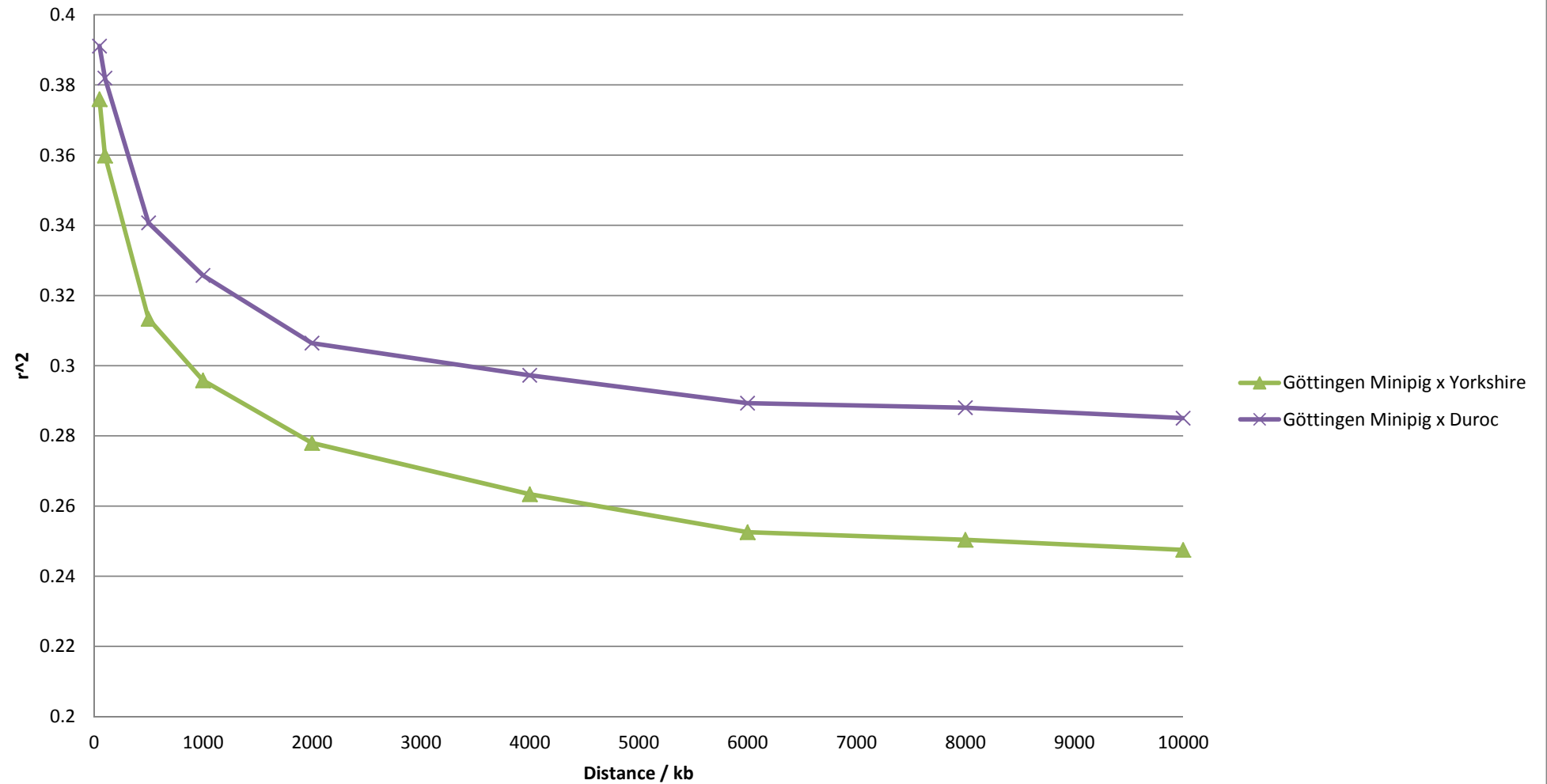

Supplement: S1 Fig — Decay of average r2 over distance calculated by the method describe by Badke et al. (2012) [56]. Average LD over short distances corresponds well to within-population LD observed previously [56]. Over longer distances, significantly stronger LD is found in the present cross which is in accordance with the LD generated by crossing different breeds. (PDF) [file pone.0137356.s001.pdf]
